# Paroxysmal Sympathetic Hyperactivity Syndrome in the Setting of Fat Emboli Syndrome Secondary to Polytrauma

**DOI:** 10.1155/2024/9888208

**Published:** 2024-05-29

**Authors:** Lauren Gould, Michael Taylor, Matthew Forestiere

**Affiliations:** ^1^ Emergency Medicine Lakeland Regional Hospital, Lakeland, Florida, USA; ^2^ Trauma/Critical Care Surgery Lakeland Regional Hospital, Lakeland, Florida, USA

## Abstract

Paroxysmal sympathetic hyperactivity (PSH) syndrome is a potentially life-threatening complication after traumatic brain injuries that results from a massive release of catecholamines in the brain. Fat embolism syndrome (FES) is a complication of long bone fractures that results in cerebral or pulmonary fat emboli. We describe PSH in the setting of cerebral FES in an adolescent female following polytrauma secondary to a motor vehicle collision to highlight the importance of rapid diagnosis and treatment of this rare complication.

## 1. Background

Paroxysmal sympathetic hyperactivity syndrome, colloquially referred to as “neuro-storming,” is a potentially life-threatening complication after traumatic brain injuries that results from a massive release of catecholamines in the brain. PSH syndrome is a clinical diagnosis made up of a constellation of signs including tachycardia, hypertension, tachypnea, diaphoresis, posturing, and fever. The patient may experience all or only some of these features, making the diagnosis challenging. One diagnostic tool that can be used to assist in the diagnosis is the paroxysmal sympathetic hyperactivity diagnostic likelihood tool as seen below in Table 1. Fat embolism syndrome is a complication of long bone fractures that result in fat emboli causing skin manifestations of petechial rash, pulmonary fat emboli that may cause respiratory failure, and cerebral fat emboli causing neurologic deficits and symptoms (cerebral fat emboli syndrome). We describe PSH in the setting of FES in an adolescent female following polytrauma that included multiple long bone fractures to highlight the importance of rapid diagnosis and treatment of this rare possibly life-threatening complication known as paroxysmal sympathetic hyperactivity syndrome.

## 2. Case

A previously healthy 15-year-old female presented as a high-level trauma alert by EMS following a motor vehicle collision. The patient was a restrained passenger in a vehicle that was a high-speed, front-end collision. There was significant front-end damage causing both airbags to be deployed. The patient arrived at the hospital with a Glasgow Coma Scale (GCS) of 15 despite losing consciousness and being amnestic to the events. She was found to have a displaced comminuted fracture of her left femur, multiple pelvic fractures, and bilateral tibia/fibula fractures, with the left side being a significantly comminuted fracture. Other injuries included a small left apical pneumothorax (less than 5%), small grade 2 liver laceration, two left rib fracture, and multiple ecchymoses. Initial imaging of CT head and cervical spine was negative upon arrival. Patient's left lower extremity was placed in traction upon admission and arrival to the intensive care unit (ICU).

Approximately nine hours after arrival, her neurological status rapidly declined to a GCS of 7 with only opening her eyes and extensor posturing to pain. She was intubated for airway protection and urgently taken for repeat CT imaging to include angiography of the brain. The head, neck, or thorax imaging did not demonstrate any worsening bleeding, new injuries, or central pulmonary embolism. Labs were unremarkable aside from leukocytosis (WBC 22.11), elevated lactic acid of 4.6, and creatinine kinase level of 3,216. The patient was not visualized to have a seizure but was loaded with Keppra and an EEG ordered.

**Figure 1:**
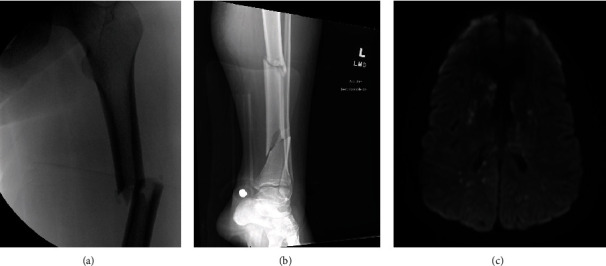
Patient imaging: (a) closed displaced left femur fracture; (b) closed displaced comminuted fracture of the left tibia; (c) MRI imaging demonstrating multiple hyperintense foci representing fat emboli.

On ICU day 2, an MRI brain/brainstem was performed. MRI revealed innumerable multifocal bilateral foci of restricted diffusion with associated areas of cytotoxic edema possibly representing fat embolism phenomenon. MRI and long bone fracture images can be seen in Figure 1. EEG monitoring was negative for seizures or epileptiform activity.

**Figure 2:**
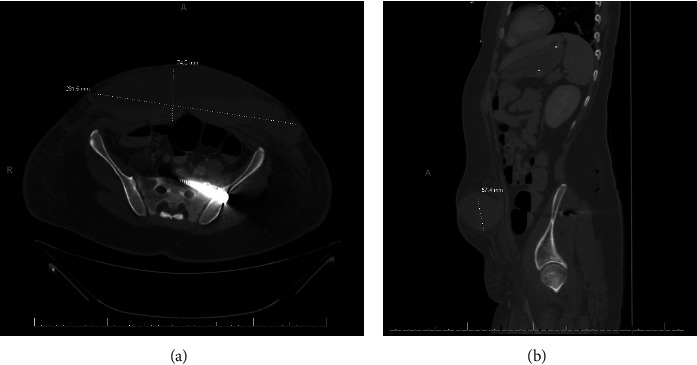
Patient imaging demonstrating an aspect of the patient's polytrauma injuries involving a ML lesion: (a) CT imaging of axial view of Morel-Lavallée hematoma measuring 74 mm × 281.5 mm; (b) sagittal view of CT imaging of Morel-Lavallée hematoma measuring 57.4 mm in height.

We considered diffuse axonal injury as an alternative diagnosis; however, the patient's original head CT was negative for intracranial hemorrhage or edema, and the subacute change in the patient's mental status made FES more likely because the fat emboli secondary to the patient's long bone fractures fit the timeline of the subacute brain injury. We also considered infection or sepsis as a possibility for the patient's change in mental status; however, initial abdominal CT did not demonstrate hollow viscous injury, and repeat CTs also did not demonstrate any intra-abdominal pathology that would cause infection, and no other infectious etiology was identified throughout the patient's workup. One of the patient's multiple abdominal/pelvic CT images can be seen in Figure 2.

On ICU day 3, an echocardiogram was performed that detected a patent foramen ovale (PFO). Left ventricle ejection fraction found to be 55-65% with no valvular lesions. She also had surgical internal fixation of her right ankle and external fixation of her left femur.

Starting on ICU day 4, she began to have spontaneous eye opening but was not tracking or following commands. Multiple episodes of suspected PSH were witnessed. The patient's polytrauma made initial diagnosis of PSH more difficult in determining if her tachycardia and hypertension were due to PSH or simply secondary to pain; however, using the PSH diagnostic likelihood tool in Table 1 allowed for confirmation of the diagnosis of PSH. The patient demonstrated tachycardia (147 beats/min), hypertension (150/99 mmHg), mechanical asynchrony with tachypnea (27 breaths/min), muscle spasms with extensor posturing, diaphoresis, and elevated temperatures up to 37.9°C. These clinical features correlated with a score of 18 on the PSH diagnostic likelihood tool (see Table 1).

She was started on propranolol 20 mg three times a day and scheduled oxycodone every 4 hours. The patient's frequency of episodes decreased over the next few days. She was deemed appropriate from both a hemodynamic and neurologic standpoint to undergo definitive fixation of her orthopedic injuries on ICU day 7.

Subsequently, her abdominal ecchymoses from her seatbelt injury were found to be acutely expanding and indurated, and CT imaging of the area revealed worsening of her Morel-Lavallée lesion from her seatbelt injury. She underwent operative evacuation of the hematoma, and two JP drains were placed at the site of the Morel-Lavallée lesion.

The patient improved neurologically with more spontaneous and coordinated movements and more consistently following commands. She was extubated successfully on ICU day 18. Aggressive physical therapy and speech therapy were initiated in the TICU due to continued slowed response and weak phonation. On day 23, the patient was transferred from the ICU to the floor for continued recovery with aggressive physical and speech therapy with goal of transition to rehabilitation care. The patient was discharged home on day 39 with outpatient orthopedic follow-up and continued home physical therapy.

## 3. Discussion

This case involved a 15-year-old healthy female who developed FES complicated by PSH following multiple traumatic closed long bone and pelvic fractures. While our patient was found to have a patent foramen ovale, imaging confirmed absence of thromboembolic phenomenon, securing the diagnosis of cerebral FES. It should be noted, as evidenced in previous literature review, that absence of PFO does not preclude development of FES as PFO was only identified in 12% of patients [2].

It is important when making the initial diagnosis of FES to consider the patient's history, physical exam, and sequence of events to distinguish from other possible diagnoses. A patient with initially no external head injuries and negative initial head CT imaging would make diffuse axonal injury less likely. In trauma patients with no source of infection identified, sepsis would also be lower on the differential. In a trauma patient with long bone fractures, an initial negative head CT and a subacute change in mental status-FES become a leading diagnosis on the differential. Early identification of PSH can prove difficult given that its clinical presentation shares similar features with multiple other pathologies commonly seen in the polytrauma patient including early sepsis, pulmonary embolism, medication effects, and withdrawal syndromes. The diagnosis of PSH after an intracranial pathology has been identified can be assisted using the paroxysmal sympathetic hyperactivity diagnostic likelihood tool. Once PSH has been identified, then appropritate treatment can be intiated.

Our patient's episodes of PSH were able to be controlled using adequate pain control medications and titrating propranolol to an appropriate dose for the patient. Seizure prophylaxis was also initiated due to cerebral edema caused by FES. Other possible treatments include steroids such as dexamethasone or methylprednisolone to reduce cerebral edema, the use of albumin that binds with the fatty acids restoring blood volumes, 5% alcohol glucose solution that can inhibit the formation of fat droplets, or the use of lipid-soluble drugs [3]. By attempting to reduce the load of fat emboli and reduce cerebral edema, episodes of PSH may be limited. However, if PSH episodes do occur, then it is important to control the sympathetic hyperactivity to limit long-term effects on the brain and body with pain control and adjuncts including propranolol and possibly gabapentin.

Overall, the patient had a positive outcome; however, therapy for PSH is patient specific, and drugs and doses may need to be adjusted to control the patient's PSH episodes on an individual level. There are also multiple suggestions for FES treatment and few suggestions on treating PSH episodes; however, no formal or specific treatment guidelines currently exist for treating either. There is a paucity of data regarding PSH as a complication of cerebral FES [3, 4]. Further investigation and studies are warranted to better guide treatment options in these challenging cases.

## 4. Conclusion

Diagnosis of FES can be difficult especially in polytrauma patients due to a broad differential diagnosis; however, using tools such as the PSH diagnostic likelihood tool as described in Table 1, the diagnosis can be made in a timelier manner. Expedited diagnosis leads to appropriate treatment and faster and better outcomes for patients. It is also important to note possible prevention of FES with early immobilization of fractures using traction. There is controversy over whether prophylactic corticosteroids can decrease the incidence of FES, but this is another preventative measure to consider [5]. FES and possible resulting PSH are important sequelae to keep in mind when managing polytrauma patients, and taking a multifactorial approach to treating these patients is important to optimize positive outcomes.

## Figures and Tables

**Table 1:** Paroxysmal sympathetic hyperactivity (PSH) diagnostic likelihood tool [1].

	Clinical Feature Scale (CFS)				Score
	0	1	2	3	
Heart rate	<100	100-119	120-139	>140	**3**
Respiratory rate	<18	18-23	24-29	>30	**2**
Systolic blood pressure	<140	140-159	160-179	>180	**2**
Temperature	<37	37-37.9	38-38.9	>39	**1**
Sweating	Nil	Mild	Moderate	Severe	**2**
Posturing during episodes	Nil	Mild	Moderate	Severe	**2**
				CFS subtotal	**12**
Severity of clinical features	Nil	0			
	Mild	1-6			
	**Moderate**	**7-12**			
	Severe	≥13			
Diagnosis likelihood tool (DLT)—1 point for each feature present					
Clinical features occur simultaneously					**1**
Episodes are paroxysmal in nature					**1**
Sympathetic over-reactivity to normally nonpainful stimuli					**1**
Features persist ≥3 consecutive days					**1**
Features persist ≥2-week postbrain injury					**0**
Features persist despite treatment of alternative differential diagnoses					**0**
Medication administered to decrease sympathetic features					**1**
≥2 episodes daily					**1**
Absence of parasympathetic features during episodes					**0**
Absence of other presumed cause of features					**0**
Antecedent of acquired brain injury					**0**
				DLT subtotal	**6**
Combined total (CFS+DLT)					
PSH diagnostic likelihood	Unlikely	<8			
	Possible	8-16			
	**Probable**	**≥17**			

Based on the PSH diagnostic likelihood tool, the patient has a Clinical Feature Scale of 12 which indicates moderate severity of clinical features and a diagnosis likelihood tool of 6. The combined total scores equal 18 which indicates that PSH is the probable diagnosis. The patient's score is calculated in bold values.

## Data Availability

Access to additional data regarding this case report can be made upon request to the authors. Some data may be restricted in order to protect patient privacy. Requests can be made to Dr. Lauren Gould at lauren.gould@mylrh.org.
